# Exploring the effects and potential of unlocked I/O-powered single board computer clusters

**DOI:** 10.1038/s41598-025-34623-x

**Published:** 2026-01-07

**Authors:** Yeongmo Lee, Dongchul Park

**Affiliations:** 1 Department of Convergence Security, Chung-Ang University, 84 Heukseok-ro, 06974 Dongjak-gu, Seoul, South Korea; 2 Department of Industrial Security, Chung-Ang University, 84 Heukseok-ro, 06974 Dongjak-gu, Seoul, South Korea

**Keywords:** Energy science and technology, Engineering, Mathematics and computing

## Abstract

Across all fields, experts strive to collect and analyze numerous data to extract meaningful insight. In response to this trend, Hadoop and Spark have emerged, and many organizations have adopted these platforms for big data storage and processing. In addition, data centers with powerful servers are constantly expanding to accommodate the increasing number of data, causing significant costs and environmental problems due to the tremendous energy consumption. Single board computer (SBC) clusters have emerged as a promising alternative for efficient computing. Most SBCs have adopted a microSD slot for data storage; thus effectively processing massive data has some limitations. However, the latest generation Raspberry Pi (RPi), model 5B provides a peripheral component interconnect express (PCIe) interface, enabling high-performance storage media, such as solid state drives (SSD). This paper extensively investigates the practicability and potential of SBCs for terabyte-scale big data processing. We build the SBC Hadoop cluster, adopting the most powerful, latest RPi 5B (8 GB of RAM) with a fast PCIe-based SSD via the PCIe interface, and perform six widely known benchmarks with a large (up to 2 TB) data size. Furthermore, this paper discusses challenges and suggestions, including the effects of input/output (I/O) throughput, central processing unit (CPU) overclocking, power supply, and trim command, which significantly affect SBC Hadoop performance. This comprehensive study concludes that integrating the enhanced computing of RPi 5B with unlocked I/O performance finally paves the way for a practical solution to real-world big data processing on SBC clusters.

## Introduction

In contemporary society, the significance of big data has increased due to its significant potential across sectors, including business, health care, finance, and government^1–3^. This exponential data growth requires efficient data processing and analysis. Apache Hadoop and Spark platforms have emerged to address these challenges, revolutionizing the market by providing scalable and efficient solutions for big data storage and processing^4^. Their advent marks a pivotal development in the field of big data analytics. Hadoop, using the Hadoop Distributed File System (HDFS) and MapReduce processing framework, offers a scalable and fault-tolerant solution for managing massive datasets. Spark provides an in-memory processing framework to accelerate data computation and has become the preferred choice for real-time analytics, stream processing, and machine learning applications^5^.

The rapidly increasing scale of data increases the need for efficient storage and processing capabilities to manage massive volumes of data. Cloud service providers, such as Amazon Web Service (AWS) and Microsoft Azure have been expanding their global data center infrastructure to reduce cloud costs^6^. Data centers consume an enormous amount of electricity to operate and cool their systems. Today, geographical features, such as a cold climate, are commonly employed as solutions to reduce data center heat generation and power consumption^7^. However, this approach is impractical for most enterprises (other than big tech companies). Introducing servers with high power efficiency for big data processing could provide a more direct solution. From this perspective, a single board computer (SBC) offers significant advantages in terms of power efficiency, making it a promising solution to financial and environmental challenges.

The SBC has significantly evolved since its inception in the 1970s, with the market experiencing substantial growth following the launch of the Raspberry Pi (RPi) controller in 2012^8^. Initially aimed at promoting computer science education, RPi has transcended its original purpose to become a versatile tool embraced by hobbyists, educators, and professionals. With each new iteration, the capabilities of RPi have been enhanced, offering more memory, better processing power, and improved connectivity. Researchers have begun investigating using RPi clusters in more challenging applications, such as big data processing and micro data centers^4^. Moreover, RPi has consistently relied on a micro secure digital (microSD) card for storage media. Due to the low performance and small capacity of microSDs, processing big data at practical, useful speeds has been challenging^9^.

With the release of the latest generation RPi (i.e., RPi 5B) in late 2023, the peripheral component interconnect express (PCIe) interface was finally adopted, providing an option to mount modern high-performance storage media other than the slow microSD. Thus far, the central processing unit (CPU) has been upgraded to a quad-core processor running at speeds of up to 2.4 GHz, the random access memory (RAM) has increased to 8 GB, and the Ethernet speed has improved to 1 Gbps.

Research on the potential use of SBCs for big data processing has been steadily conducted. However, conventional SBCs have had limitations in terms of integration with other hardware. To mitigate structural constraints on storage performance, some studies have adopted Network Attached Storage (NAS) or Universal Flash Storage (UFS) cards, but higher-performance storage media are still required for practical big data processing. With the introduction of a PCIe interface, the RPi 5B can be regarded as the first SBC model to effectively break free from storage media constraints. In this study, the concrete performance of big data processing in an SBC environment combined with an SSD is presented, and the potential of SBCs for big data processing is demonstrated.

This study investigates the feasibility of processing big data using the SBC cluster by natively integrating an M.2 solid state drive (SSD) into the latest generation RPi (i.e., RPi 5B with 8 GB of RAM) via a PCIe 3.0 ($\times$1) interface by adopting a new hardware-attached-on-top (HAT) board. This hardware configuration of RPi is currently the most powerful for big data processing. First, this study extensively measures the performance of RPi 5B individually to investigate its computational capability enhancement compared to the previous generation of RPi (i.e., RPi 4B) released in 2019. Then, an SBC Hadoop cluster is built, consisting of one RPi 5B as a master node and eight RPi 5B units as worker nodes, and a series of representative benchmarks, including WordCount, TeraGen and TeraSort, Pi computation, Grep, and TestDFSIO, are used to evaluate the performance of RPi Hadoop and Spark clusters. In addition, for a more objective evaluation, the RPi 5B cluster is compared with the newest generation of desktop computer, providing informative insight into the possibility of real-world big data processing using SBC Hadoop clusters.

Furthermore, this study examines the storage media effect on the big data processing platforms by comparing the differences between the fastest microSD and PCIe-based SSD storage options available for the RPi 5B. Last, a discussion and the findings are presented, covering CPU overclocking (2.4 to 3.0 GHz) to discuss the influence of CPU performance, the PCIe bandwidth (PCIe 2.0 to 3.0) to study the influence of storage bandwidth, the trim command to explore the garbage collection problem of flash memory, the power supply to verify the correlation between the power supply amount and RPi performance, and multiple application execution to evaluate the parallel computing capability.

To our knowledge, this study is quite comprehensive, covering several problems, findings, and suggestions for the feasibility of SBCs in real-world big data processing by adopting the most powerful RPi configurations. This study concludes that the latest significantly improved computing capability of RPi 5B with the fastest modern SSD storage media offers a practical solution for small, terabyte-scale big data processing on SBC Hadoop clusters. Unlike previous RPis, the data size is no longer a limitation because the RPi 5B Hadoop cluster effectively and stably expands processing and storage capabilities. Overall, the PCIe interface of RPi 5B is extremely beneficial.

The main contributions of this paper are as follows:

- **Big data processing on a powerful SBC without an I/O Bottleneck** The existing SBC-based big data processing has limitations due to the performance of the SBC and the storage media constraints. The latest M.2 SSDs (500 GB) are directly connected to each SBC node via the PCIe interface, rather than other slower I/O interfaces, such as a universal serial bus (USB) port to overcome the bottleneck in SBC-based big data processing. Thus, each SBC node improves storage performance by an average of 7$\times$ faster for reading and 16$\times$ faster for writing compared to one of the fastest microSD cards on the market. This study is the first to configure the strongest RPi 5B node to explore the possibility of big data processing on the SBC Hadoop cluster comprehensively using an terabyte-scale dataset size (up to 2 TB; Section Raspberry Pi cluster performance).
- **Challenges and predicting the future** Most existing studies have primarily aimed to measure the experimental performance of each SBC Hadoop cluster because the previous SBC nodes did not have sufficient computing capabilities. Thus, these studies have focused on running Hadoop benchmarks. This study extensively discusses diverse challenges and findings that significantly affect SBC node performance. For example, a higher I/O throughput (PCIe 2.0 vs. 3.0) could not be fully utilized by RPi 5B due to the unexpected CPU bottleneck, even though its CPU performance had substantially improved, which was verified by the CPU overclocking experiments (2.4 vs. 3.0 GHz). This finding implies that the I/O throughput expansion of future SBCs must consider CPU performance improvement accordingly. In addition to this suggestion, several challenges and experiments offer insight into the potential performance of future SBC models (Section Discussion).
- **Extensive scale-Out evaluation** We evaluate six well-known benchmarks for Hadoop and Spark. Each benchmark is conducted by varying the node count from one to eight with various data sizes. These comprehensive performance evaluations offer insight into several factors, such as the hardware improvement effects and performance trends. We also measure the diverse performance metrics (CPU, network, storage media performance, and power consumption) of individual RPi models, 4B and 5B, investigating the relationship between the performance improvement of the individual node and the cluster. In the comparison of the performance of the SBC Hadoop cluster to that of the desktop computer, the Hadoop performance offers insight into the future possibilities and opportunities for the SBC Hadoop cluster for real-world big data processing (Sections Individual Raspberry Pi performance and Raspberry Pi cluster performance).

The remainder of this paper is organized as follows. Section Background knowledge provides an overview of RPi and SSD. Next, Section Related work presents the related studies. Then, Sections Individual Raspberry Pi performance and Raspberry Pi cluster performance present a variety of experimental results and analyses and Section Discussion discusses the diverse challenges. Finally, Section Conclusion concludes the work.

## Background knowledge

### Raspberry Pi

The RPi is a series of SBCs developed by the RPi Foundation to promote the study of computer science^10^. Designed to make computing affordable and available to everyone, the RPi quickly gained popularity and has transformed into a powerful and versatile platform used in schools, businesses, and projects worldwide. The first model, RPi Model B (RPi B), was released in February 2012, and since then, it has significantly evolved. The RPi B features a 700 MHz single-core ARM processor with 256 MB of RAM, targeting educational use to promote computer science. Launched in 2015, RPi 2B offers a quad-core processor and 1 GB of RAM. Starting in 2016, RPi 3B introduced built-in Wi-Fi and Bluetooth. In 2019, RPi 4B included substantial upgrades, initially including up to 4 GB of RAM (which was recently upgraded to 8 GB), a USB 3.0, and dual 4K display support^11^.

The fifth and latest generation in the series, RPi 5B was again significantly upgraded over its predecessor. It featured a new Broadcom BCM2712 system-on-a-chip with four ARM Cortex-A76 cores clocked at 2.4 GHz, offering two times faster computing performance than its predecessor, the RPi 4B. In addition, RPi 5B was enhanced with a VideoCore VII graphics processing unit (GPU) for better graphics and introduced a new RP1 chip for improved I/O handling. A PCIe interface provides expanded customization potential, such as a non-volatile memory express (NVMe) SSD and 10 Gb for networking. The RPi 5B maintains its legacy of offering high performance at an affordable price. Each iteration has built on the success of its predecessors, cementing the reputation of RPi as a powerful and versatile platform for a wide range of applications^12^.

### Storage connection interface

Recently, SSDs have rapidly gained popularity due to their improved performance, reliability, and price and have gradually been widely adopted in personal computers and servers^13,14^. Early SSDs were connected to the motherboard using the serial advanced technology attachment (SATA) interface, the same interface used by traditional hard disk drive (HDD)^15^.

However, the SATA interface was initially designed for conventional HDDs, making it difficult to exploit the SSD speed fully. An NVMe SSD connected to the PCIe interface has emerged to eliminate this bottleneck^16^. The PCIe interface resolves the limitations of conventional interfaces in terms of bandwidth and scalability. The number of lanes ($\times$1, $\times$4, $\times$8, $\times$16, etc.) allows bandwidth adjustments as needed. Although the SATA III SSD is limited to a maximum transfer rate of 6 GT/s, the PCIe 3.0 $\times$4 NVMe SSD offers transfer rates of up to 32 GT/s, and the PCIe 4.0 $\times$4 NVMe SSD offers transfer rates of up to 64 GT/s^17^. The PCI Special Interest Group recently released the PCIe 7.0 specification (up to 128 GT/s per lane), and plenty of room exists to improve SSD performance.

The RPi 5B officially supports the PCIe interface. Although the PCIe connection of the RPI 5B is certified for PCIe 2.0 speeds (5 GT/s), PCIe 3.0 (8 GT/s) speeds can be forced by configuring the PCIe option. The previous generation of RPi (i.e., RPi 4B) was able to connect an SSD indirectly using a USB 3.0 port. However, the RPi 5B is the first generation to provide a native PCIe interface on the board, allowing a direct PCIe SSD connection. The USB 3.0 port, with a maximum bandwidth of 5 Gbps, limits the ability to apply the SSD performance fully, whereas PCIe allows scalability depending on the version and number of lanes. With the continuous advancement of big data and artificial intelligence (AI), the SBC must increasingly handle high bandwidths. Thus, the next generation RPi is expected to upgrade the PCIe version or increase the lane count to improve SSD employment.

## Related work

Many researchers have studied low-powered SBC clusters in diverse fields, such as edge computing^18^, cloud^19^, database^20^, blockchain^21^, AI^22^, and cryptography^23^. Among the many papers on SBC clusters, this section focuses on SBC-based big data processing because it is directly associated with this research.

Adnan et al.^9^ built a cluster using Banana Pi M3 (octa-core 2 GHz CPU with 2 GB of RAM and a 1 Gbps Ethernet) and evaluated the big data processing performance with two storage types: microSD and NAS. They aimed to address the limitations of the microSD as the primary storage media for the SBC and proposed the NAS as an alternative. They evaluated the performance of Hadoop using the TeraSort benchmark for single and multinode configurations. Although the performance difference between the two storage media options was just 2 seconds for a single node and 7 seconds for multiple nodes, they suggested the NAS due to its lower I/O wait time. However, they adopted a small data size (up to 2 GB for a single node and up to 4 GB for multiple nodes) for big data and focused on the primary storage limitations of SBCs.

Qureshi and Koubaa^24^ explored the energy efficiency of SBC clusters for big data applications. They built an ARM-based RPi 2B cluster and an Odroid XU-4 cluster and evaluated the performance of these clusters. Their study claimed that SBC-based clusters are generally energy efficient, whereas the cost-to-performance ratio depends on the workload. For a smaller workload, the XU-4 cluster, comprising 20 Odroid XU-4 in a cluster, is more cost-effective and power-efficient than the RPi cluster. For high-intensity tasks, such as TeraGen and TeraSort, the XU-4 cluster consumes significantly more energy. They concluded that the RPi cluster consistently underperformed on all benchmarks. However, they employed an older generation RPi (i.e., RPi 2B) with a 900 MHz quad-core ARM Cortex-A7 CPU and 1 GB of RAM, which did not have sufficient computing capabilities to run big data applications.

Lee et al.^25^ conducted an in-depth investigation into the challenges and potential of big data processing using an RPi 4B cluster. They constructed a five-node RPi 4B (quad-core 1.5 GHz CPU with 4 GB of RAM) cluster. This study focused on the effect of storage media performance in the RPi cluster using three portable storage media cards with various performance characteristics: a typical microSD, the fastest microSD, and UFS cards. The study claimed that faster storage media significantly improve SBC cluster performance, demonstrating a 1.3$\times$ to 7.07$\times$ performance improvement. They concluded that the RPi 4B cluster exhibited the potential to process actual big data.

Unlike these studies, this study aims to explore the possibility of *real-world* big data processing on the most powerful RPi Hadoop clusters by adopting terabyte-scale big data (up to 2 TB) and the most powerful RPi Hadoop cluster. Moreover, a variety of challenges and extreme experiments are provided for informative insight.

## Individual Raspberry Pi performance

### Hardware configurations

#### Raspberry Pi: 4B vs. 5B

**Table 1:** Specifications for Raspberry Pi models 4B and 5B.

	RPi 4B	RPi 5B
CPU	ARM Cortex-A72	ARM Cortex-A76
	@ 1.5 or 1.8 GHz (4 cores)	@ 2.4 GHz (4 cores)
RAM	1,2,4, or 8 GB LPDDR4	4 or 8 GB LPDDR4X
Ethernet	Native Gigabit Ethernet	
Bluetooth	Bluetooth 5.0	
USB	USB 3.0 $\times$ 2 + USB 2.0 $\times$ 2	
GPU	VideoCore VI @ 600 MHz	VideoCore VII @ 1 GHz
PCIe	-	PCIe 2.0 $\times$ 1
SDIO speed	Up to 43 MB/s	Up to 89 MB/s
Power consumption	3.7 W(idle), 7.0 W(full-load)	5.5 W(idle), 10.3 W(full-load)
Release	June 2019	October 2023
Price	$35(1 GB), $45(2 GB),	$60(4 GB), $80(8 GB)
	$55(4 GB), $75(8 GB)	

First, we compared the capability of the individual RPi 4B and 5B. Table 1 presents the hardware specifications of both models, where RPi 5B displays performance improvements over RPi 4B, especially on the CPU. The number of CPU cores remains the same at four, but the clock speed increased from 1.5 to 2.4 GHz. The initial RPi 4B models with 1, 2, and 4 GB of RAM provided a 1.5 GHz CPU clock speed and only the recently released RPi 4B with 8 GB of RAM had a 1.8 GHz CPU clock speed. An identical RAM size (8 GB) was selected for a fair evaluation. The most critical part is the PCIe interface of RPi 5B. The single-lane PCIe 2.0 interface was provided. However, we can easily switch the default PCIe version of RPi 5B from PCIe 2.0 to 3.0 via boot configurations, causing a substantial 2$\times$ I/O throughput improvement from 400 to 800 MB/s (Section PCIe Version: 2.0 vs. 3.0).

#### Storage media: Fastest MicroSD vs. PCIe-based SSD

**Table 2:** Characteristics of the microSD and solid state drive (SSD) employed for our experiments.

	microSD	NVMe SSD
Model code	MB-MD256SA	SHGP31-500GM-2
Model name	Samsung PRO plus	SK Hynix GOLD P31
Capacity	256 GB	500 GB
Form factor	microSD (SDXC)	M.2 (NVMe)
Read(seq.)	Up to 180 MB/s	Up to 3,500 MB/s
Write(seq.)	Up to 130 MB/s	Up to 3,200 MB/s
Read(rand.)	Minimum 4,000 IOPS (A2)	Up to 570,000 IOPS
Write(rand.)	Minimum 2,000 IOPS (A2)	Up to 600,000 IOPS

Note: Performance numbers excerpted from their product specifications.

Two types of storage media (microSD and NVMe SSD) were employed to investigate the effect of the NVMe SSD directly connected to RPi via PCIe interface. Since the first generation of RPi, the microSD has been used as primary storage media, and even the latest RPi 5B supports a single microSD card slot. An M.2 HAT board is required to attach an NVMe SSD to RPi 5B via the PCIe interface. Because SSD is an additional storage media, it must be mounted to be recognized by the operating system (OS) *(e.g., mount /dev/nvme0n1 /mnt/nvme0n1)*. Table 2 presents the microSD (Samsung PRO Plus) and SSD (SK Hynix GOLD P31) specifications. Currently, Samsung PRO Plus is recognized as one of the fastest microSD cards^26^. Moreover, SK Hynix GOLD P31 is one of the best-selling PCIe 3.0-based NVMe SSDs worldwide^27^.

#### Peripheral components and accessories

**HAT board:** The HAT board is an essential component for connecting the RPi 5B with an SSD, and the Pinberry Pi Hat Drive Bottom was used in this experiment. The HAT board connects the RPi 5B and SSD via an FPC ribbon cable, which also provides power. It supports both PCIe versions 2 and 3, and in this experiment, a HAT board compatible with the 2280 M.2 SSD form factor was employed.

**Cooling fan:** Both the RPi 4B and RPi 5B use cooling fans officially supported by Raspberry Pi. The RPi 4B fan is connected via a GPIO header, whereas the RPi 5B is designed with cooling in mind and can be connected through a four-pin fan header. The maximum airflow of the RPi 4B fan is 1.4 CFM, while that of the RPi 5B fan is 1.09 CFM.

**Power supply:** The power supply is also officially supported by Raspberry Pi. However, the power requirements differ between the RPi 4B and RPi 5B. The RPi 4B is provided with a 15W power supply, whereas the RPi 5B, due to increased power consumption, requires a 27W power supply. Using an RPi 4B power supply for the RPi 5B results in performance degradation. Details about power and performance will be discussed in Section Discussion.

### Power consumption

**Table 3:** Power consumption of Raspberry Pi (RPi) model 4B and 5B in idle and stress modes.

Modes	RPi 4B	RPi 5B
Idle mode	3.75 W	5.54 W
Stress mode	7.06 W	10.31 W

The Bplug S01 power meter was employed to measure the power consumption of each RPi model (4B and 5B). In idle mode, the power consumption was measured for 1 hour without running programs. In stress mode, the CPU was subjected to the maximum load for 1 hour *(stress –cpu 4 –timeout 3600)*. According to Table 3, both models consumed 1.8$\times$ more power in the stress mode than in the idle mode. Compared to RPi 4B, RPi 5B consumed an average of 1.4$\times$ more power in both modes due to the upgraded CPU performance. Therefore, RPi 4B uses a power supply capable of delivering 20 W, whereas RPi 5B employs a 27 W power supply to ensure sufficient power.

### CPU performance

**Table 4:** CPU performance (events per second) of RPi model 4B and 5B based on the number of threads.

Threads	1	2	4	8	16
RPi 5B (8 GB)	24,699.51	49,408.40	98,699.69	98,741.23	98,690.85
RPi 4B (8 GB)	14,122.55	28,345.28	56,644.43	56,619.70	56,744.83

The CPU performance of RPi 4B and 5B was measured using the *sysbench* tool by varying the thread count. Although both models have the same core count (four cores), RPi 5B (2.4 GHz) has a higher clock speed than RPi 4B (1.8 GHz). The number of threads increases from 1 to 16 *(sysbench –num-threads=1 –test=cpu –cpu-max-prime=2000 run)*. As in Table 4, the performance doubles to four threads and does not increase afterward because both RPi 4B and 5B are a quad-core models. The RPi 5B displays an average of 1.74$\times$ better performance than the RPi 4B.

### Network performance

**Table 5:** Network bandwidth of RPi 4B and 5B.

Network Interface	RPi 4B	RPi 5B
Ethernet	936 Mb/s	937 Mb/s
Wi-Fi 2.4 GHz	52.1 Mb/s	52.7 Mb/s
Wi-Fi 5.0 GHz	90.4 Mb/s	97.5 Mb/s

In a cluster, the network performance significantly affects the overall performance because cluster nodes communicate with each other intensively^28^. The RPi 4B and 5B have 1 Gbps Ethernet and dual-band 802.11ac wireless network interfaces. Table 5 presents their network bandwidth. An open-source network performance measurement tool called *iPerf3* was employed to test the on-board raw network throughput. The *iPerf3* measures and analyzes the network performance, including testing the bandwidth, latency, packet loss rate, and other aspects of network connections. In addition, *iPerf3* requires two performance measurement steps. First, we must open a socket on the server where the performance is measured *(iperf3 -s)*. Then, on the client sending data, the target server IP is referred to transmit data *(iperf3 -c 192.168.45.99 -i 1)*. A noticeable performance discrepancy was not found because both models have the same network specifications. Therefore, the wired network throughput of both models indicates an average performance close to a maximum theoretical throughput of 1 Gbps.

### Storage media performance

**Table 6:** Storage media performance (MB/s) for microSD and SSD on RPi 4B and 5B.

Storage media	Node	Record size	Read (seq.)	Write (seq.)	Read (rand.)	Write (rand.)
Samsung PRO Plus	RPi 4B	4 KB	9.14	3.62	7.14	3.40
		512 KB	37.90	31.79	37.93	31.64
		16 MB	43.29	34.38	43.40	34.18
	RPi 5B	4 KB	25.71	8.63	25.02	9.19
		512 KB	87.70	51.41	87.48	51.88
		16 MB	88.07	52.22	88.30	48.84
SK Hynix GOLD P31	RPi 5B	4 KB	201.95	134.01	169.48	174.42
		512 KB	798.30	742.66	717.13	751.87
		16 MB	869.80	814.47	873.48	800.23

Note: RPi 4B does not have a PCIe interface; hence, PCIe SSD experiments are omitted.

**Table 7:** 4K random read and write performance (IOPS) for the microSD and SSD on RPi 4B and 5B.

Storage media	Node	4K Rand. Read	4K Rand. Write
Samsung PRO Plus	RPi 4B	1,828	870
	RPi 5B	6,405	2,353
SK Hynix GOLD P31	RPi 5B	17,787	44,652

The *iozone* benchmark tool was employed to measure file system performance. The sequential and random read/write performance for Samsung PRO Plus (representing the fastest microSD card) and SK Hynix GOLD P31 (representing the most powerful PCIe 3.0 SSD) on RPi were measured using *iozone*. The total file size was set to 100 MB and the record size to 4 KB (a small I/O unit), 512 KB (a medium I/O unit), and 16 MB (a large I/O unit) to test the storage performance *(iozone -e -I -a -s 100M -r 4k -r 512k -r 16M -i 0 -i 1 -i 2)*.

In Table 6, the microSD and NVMe SSD displayed weak performance at the 4 KB record size due to the significantly reduced efficiency of I/O operations when accessing data in very small blocks. As the record size increased from 4 KB to 16 MB, the read and write performance noticeably improved. For instance, the sequential read performance of RPi 5B increased to 3.42$\times$ (microSD) and 4.31$\times$ (NVMe SSD). Similarly, the sequential write performance of RPi 5B improved by an average of 6.05$\times$ (microSD) and 6.07$\times$ (NVMe SSD).

The CPU performance also significantly affects the storage performance. The performance of the microSD card in RPi 5B improved substantially compared to RPi 4B (e.g., under 4 KB of the record size, 2.81$\times$ for sequential read, 2.38$\times$ for sequential write, 3.5$\times$ for random read, 2.7$\times$ for random write). Although the CPU performance of RPi 5B noticeably improved, the maximum performance of the microSD card can still be improved. This finding implies that the next generation of RPi might saturate microSD card, particularly regarding the write performance, by further upgrading the CPU performance.

The PCIe 3.0 theoretically provides approximately 984 MB/s of bandwidth per lane. The NVMe SSD (SK Hynix GOLD P31) can provide up to 3.5 GB/s for a read and 3.2 GB/s for a write with four PCIe 3.0 lanes. However, due to the constraint (i.e., a single PCIe lane) of RPi 5, the actual performance of this NVMe SSD is limited to the bandwidth of the single PCIe 3.0 lane. Table 6 and 7 present the NVMe SSD performance on RPi 5B. For the 16 MB record size, the SSD dominates the fastest microSD card by an average of 9.8$\times$ (read) and 15.98$\times$ (write). Currently, considering that just one-fourth of the full performance of the SSD is employed in RPi 5B, we could achieve even greater SSD performance if more PCIe lanes were added or if the PCIe version were upgraded in the future.

## Raspberry Pi cluster performance

### Experimental setup

#### Cluster configurations

**Fig. 1:**
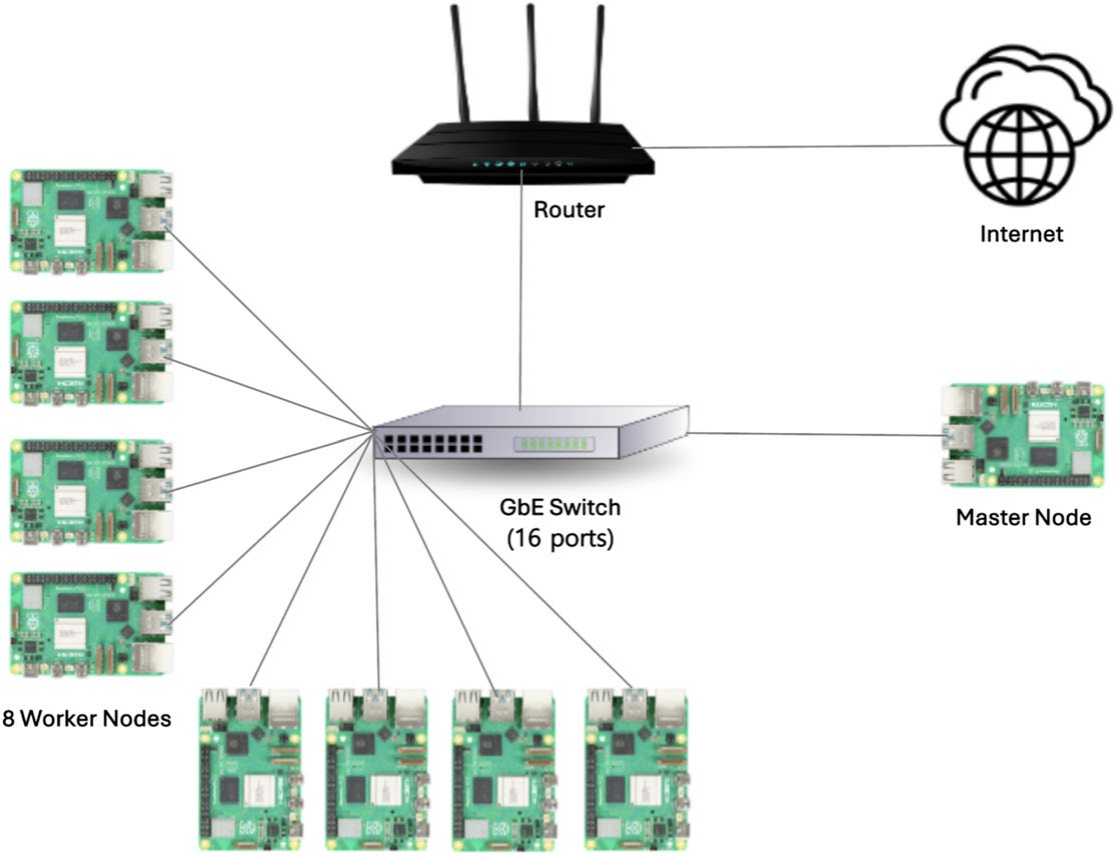
RPi 5B cluster architecture with one master node and eight worker nodes.

**Table 8:** Cluster configurations of RPi 4B and 5B.

	RPi 4B Cluster	RPi 5B Cluster
CPU	ARM Cortex-A72	ARM Cortex-A76
	@ 1.8 GHz (4 cores)	@ 2.4 GHz (4 cores)
RAM	8 GB LPDDR4	8 GB LPDDR4X
Storage	256 GB microSD	256 GB microSD, 500 GB NVMe SSD
Cluster Storage	Up to 2 TB	Up to 2 TB (microSD) or 4 TB (SSD)
Master node no.	1 (designated for a namenode)	
Worker node no.	1, 2, 4 or 8	
Network	1 Gigabit Ethernet RJ45 Jack, GbE Switches (16 ports)	
OS	Ubuntu Server 23.10, 64-Bit	
Software	Apache Hadoop V3.3.6, Spark V3.5.0	

Figure 1 illustrates the RPi Hadoop cluster architecture, consisting of eight worker nodes and one master node, all connecting to a 1 GbE network switch. Two RPi clusters (RPi 4B and 5B) were built for a more objective comparison. Table 8 lists the configurations of the RPi 4B and 5B clusters. A single master node was designated as the namenode. We increased the worker node count to 1, 2, 4, and 8, expanding the total cluster storage capacity accordingly (up to 2 TB for the RPi 4B cluster and 4 TB for the RPi 5B cluster). Ubuntu Server 23.10 (64-bit) was installed, as it is supported by the RPi Foundation as a general-purpose OS. Apache Hadoop (v3.3.6) and Spark (v3.5.0) were adopted for the experiments.

#### Hadoop and spark configurations

**Table 9:** Hadoop configurations.

mapred-site.xml	Value
yarn.app.mapreduce.am.resource.mb	1536 (default)
yarn.app.mapreduce.am.resource.cpu-vcores	1 (default)
mapreduce.map.memory.mb	1024 (default)
mapreduce.reduce.memory.mb	1024 (default)
mapreduce.map.cpu.vcores	1 (default)
mapreduce.reduce.cpu.vcores	1 (default)
mapreduce.job.reduces	2

hdfs-site.xml	Value
dfs.replication	1
dfs.namenode.name.dir	/hdfs/namenode (microSD) or
	/mnt/nvme/hdfs/namenode (SSD)
dfs.namenode.data.dir	/hdfs/datanode (microSD) or
	/mnt/nvme/hdfs/datanode (SSD)

yarn-site.xml	Value
yarn.nodemanager.resource.memory-mb	8192 (default)
yarn.nodemanager.resource.cpu-vcores	8 (default)
yarn.scheduler.minimum-allocation-mb	1024 (default)
yarn.scheduler.maximum-allocation-mb	4096 (default)
yarn.scheduler.minimum-allocation-vcores	1 (default)
yarn.scheduler.maximum-allocation-vcores	32 (default)
yarn.nodemanager.vmem-pmem-ratio	2.1 (default)

**Table 10:** Spark configurations.

spark-defaults.conf	Value
spark.master	yarn
spark.executor.instances	1, 2, 4 or 8
spark.executor.cores	2

Tables 9 and 10 list Hadoop and Spark configurations. The number of reducers and replications in Hadoop and the number of instances and cores in Spark were adjusted for more effective evaluations. Other parameters were not changed to minimize any factors that could affect the experimental results.

Hadoop with a single reducer (by default) causes inefficient operations and performance. Thus, we set it to two, considering performance and reliability. The Hadoop replication factor has a trade-off between storage consumption and reliability. We set the replication count to one to evaluate an terabyte-scale dataset.

By default, Spark sets the number of instances to two, meaning even with four nodes, only two nodes are operating. This implies that a four-node cluster and a two-node cluster perform identically. Thus, according to the node counts, this option is appropriately adjusted to match the number of nodes. In addition, the number of cores is set to two considering performance and stability.

#### Input data and method

Public data (2006.csv, 678 MB) from the American Statistical Association (ASA) were employed for an objective evaluation^29^. These data include flight arrival and departure information for every commercial aircraft operating in the United States between October 1987 and April 5, 2008. We created datasets up to 32 GB (i.e., from 1 GB to 32 GB) to investigate the performance trends and 2 TB for terabyte-scale experiments. We did not manipulate the data and only appended the file to reach the desired size. Representative benchmarks, such as WordCount, TeraGen/TeraSort, Grep, Pi computation, and TestDFSIO, were performed five times each with Hadoop and Spark. The variability across runs is presented using 95% confidence intervals in all corresponding figures. The microSD and NVMe SSD were employed to explore the influence of the storage media performance. In addition, the benchmarks were assessed using various cluster sizes by varying the cluster node count from one to eight.

### Experimental results and analyses

#### WordCount

**Table 11:** Intermediate data volume (GB) generated under the Hadoop and Spark WordCount benchmarks on RPi 5B cluster.

Data size	1 GB	2 GB	4 GB	8 GB	16 GB	32 GB
Hadoop	1.8	3.6	10.5	25.2	58.3	126.2
Spark	1.3	2.1	3.4	5.7	11.1	20

**Fig. 2:**
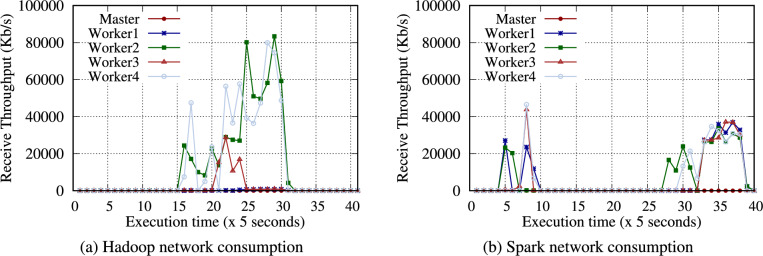
Network consumption for each node under the WordCount benchmark on an RPi 5B cluster with four worker nodes.

**Fig. 3:**
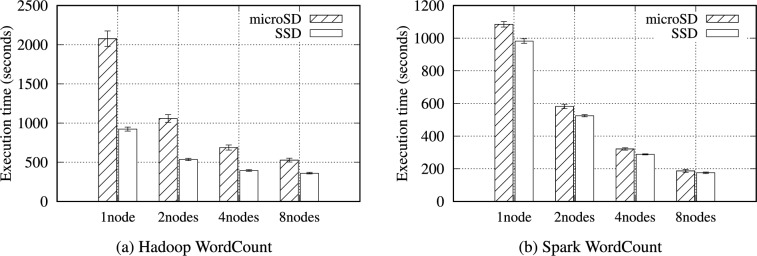
Execution time for the Hadoop and Spark WordCount benchmarks with 16 GB of data on the RPi 5B cluster.

WordCount calculates the frequency of each word. Hadoop splits the input data into multiple blocks and performs map operations on each block in parallel. The map operation reads the input data and generates intermediate data (i.e., key (word) and value (count) pairs). After the map phase, Hadoop performs a shuffling step to send the mappers’ intermediate (key, value) pairs to the Reducers. The reducer takes the list of values associated with each key, aggregates these values, and produces the result.

Spark transforms the input data into a resilient distributed dataset (RDD) and splits each line into words to create a collection of words. The data are distributed across multiple worker nodes, and each worker processes them in parallel. Spark uses the map operation of the RDD to convert each word into a key-value pair of the form (word, 1). Finally, the *reduceByKey* operation aggregates the values for the same key (i.e., the same word).

Table 11 presents the intermediate data volume written to storage for the Hadoop and Spark WordCount benchmarks, offering insight into the workload characteristics of WordCount. The intermediate data were measured using the trim command (*fstrim* in Linux) supported by the OS. Section Discussion discusses the effects of the trim command further. WordCount generates intermediate data during the map phase. With 32 GB of input data, Hadoop and Spark produced 126.2 GB (3.94$\times )$ and 20 GB (0.63$\times$) respectively of the intermediate data. Spark processes the data in memory, resulting in significantly less intermediate data than Hadoop. Generating a large volume of intermediate data suggests that the performance depends more on the storage and network performance. In Fig. 2, the network consumption of Hadoop is noticeably higher than that of Spark under the WordCount benchmark because the intermediate data in each node are transferred to the node with reducers.

Figure 3 presents the total execution time of the WordCount benchmark on the RPi 5B cluster. As the node count increases, each execution time decreases for Hadoop and Spark. In addition, the NVMe SSD performs noticeably faster than the microSD (particularly under the Hadoop WordCount) because it generates significantly more intermediate data than Spark WordCount. Exceptional I/O performance of the NVMe SSD influences the Hadoop WordCount performance. For instance, the SSD performs faster than the microSD by an average of 2.25$\times$ and 1.98$\times$ under a single node and two nodes, respectively.

Tables S24 and S25 present the experimental results of WordCount on Hadoop and Spark with diverse configurations. As the data size increases from 1 to 32 GB, the total execution time also increases almost linearly. Similarly, as the node count doubles, WordCount for both Hadoop and Spark improves by an average of 1.57$\times$ and 1.78$\times$, respectively.

**Table 12:** Intermediate data volume (MB) generated on the Hadoop and Spark TeraGen benchmarks on RPi 5B cluster.

Data size	1 GB	2 GB	4 GB	8 GB	16 GB	32 GB
Hadoop	199	208.8	198.9	268.5	91.9	152.3
Spark	484.3	277.1	353.5	551.5	326.1	366.2

**Fig. 4:**
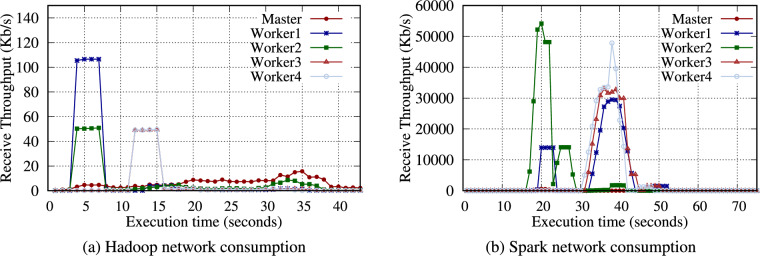
Data throughput received by each node when running the TeraGen benchmark on an RPi 5B cluster with 4 worker nodes.

#### TeraGen and TeraSort

The TeraSort benchmark sorts large datasets as quickly as possible. This benchmark consists of three primary components: TeraGen, TeraSort, and TeraValidate. TeraGen generates random data, and TeraSort rearranges the generated data. Finally, TeraValidate verifies that the sorting was performed correctly.

**TeraGen**: Hadoop TeraGen randomly generates data records comprising unique keys and values during the map phase. Then, each mapper directly writes the data to the HDFS (no reduce phase is required). Spark TeraGen creates an empty RDD with the specified number of partitions, determines the amount of data to generate in each partition, and generates random data. As in Table 12, the workload of TeraGen generates almost no intermediate data because it only needs to write to the storage media. Therefore, as shown in Fig. 4, no data are transferred between nodes during the reduce phase.

**Fig. 5:**
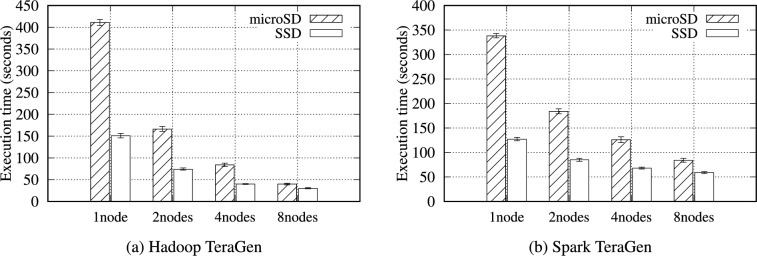
Execution time for the Hadoop and Spark TeraGen benchmarks with a 16GB data size on the RPi 5B cluster.

Figure 5 presents the total execution time for the TeraGen benchmark. The difference in performance between the microSD and SSD was more pronounced because the TeraGen consists entirely of write workloads. Under Spark WordCount benchmark with 16 GB of data on a single node, the performance gap between the microSD and SSD was 1.1$\times$, whereas it was 2.66$\times$ for TeraGen. TeraGen must write data to the storage media; thus, it does not benefit from an in-memory processing mechanism. Therefore, as the data size increases, their performance gap also increases. Table S26 and Table S27 demonstrate this. For example, performance gap between SSD and microSD increases from 1.02$\times$ (1 GB) to 3.01$\times$ (32 GB) under Hadoop TeraGen.

**Table 13:** Intermediate data volume (GB) generated during the Hadoop and Spark TeraSort benchmarks on RPi 5B cluster.

Data size	1 GB	2 GB	4 GB	8 GB	16 GB	32 GB
Hadoop	1.6	3	9.2	24.1	55.5	134.3
Spark	1.4	2.8	4.9	9.4	17.6	34.4

**TeraSort**: Hadoop TeraSort sorts the data created by TeraGen. Mappers read input data blocks and convert them into key-value pairs (intermediate data). During the shuffle and sort phase, the data are sorted by keys. Finally, reducers generate the sorted final output. Spark reads the data in RDD form, redistributes the data into a new number of partitions, and sorts it by keys in each partition.

**Fig. 6:**
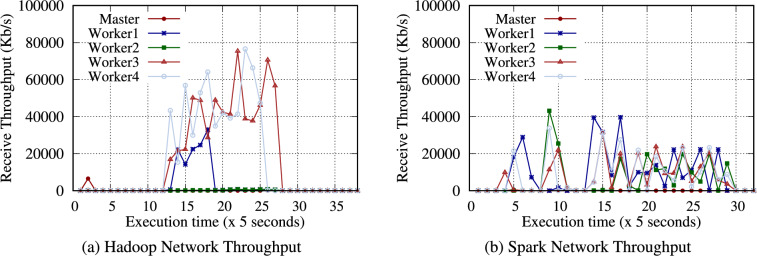
Data throughput received by each node when running the TeraSort benchmark on an RPi 5B cluster with 4 worker nodes.

Table 13 presents the intermediate volume of data for Hadoop and Spark TeraSort. Similar to WordCount, a significant amount of intermediate data was observed. Notably, Hadoop generated 4.2$\times$ intermediate data for input data. TeraSort generated more intermediate data than WordCount. In Fig. 6, Hadoop TeraSort intermediate data were transferred to the nodes designated as reducers with a high network consumption. In contrast, the intermediate data for Spark TeraSort were continuously transferred between nodes after reading data.

**Fig. 7:**
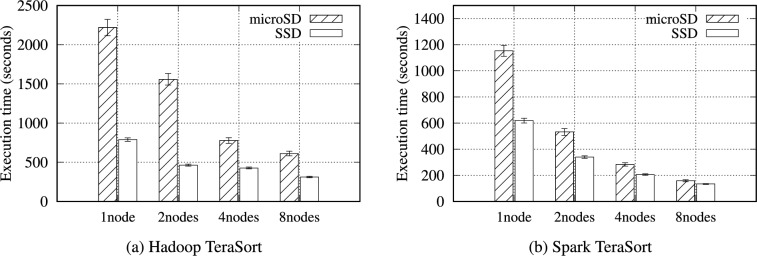
Execution time for the Hadoop and Spark TeraSort benchmarks with a 16GB data size on the RPi 5B cluster.

**Fig. 8:**
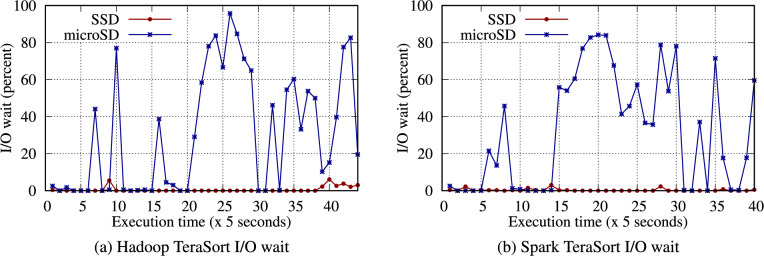
I/O wait time for the Hadoop and Spark TeraSort benchmarks on the RPi 5B Cluster.

Figure 7 presents the total execution time for Hadoop and Spark TeraSort. Tables S28 and S29 provide detailed TeraSort experimental results across different configurations. The performance discrepancy is most significant between the microSD and NVMe SSD in TeraSort compared with other benchmarks, as shown by the difference in I/O wait in Fig. 8. For instance, Spark TeraSort has a 2.59$\times$ performance gap on a single node with 32 GB. In contrast, Spark WordCount has only a 1.1$\times$ performance gap because TeraSort generates more intermediate data and has the same output data size as the input data size. Thus, TeraSort writes an even larger volume of data to storage than WordCount, accounting for the differences.

**Table 14:** Intermediate data volume (MB) generated under the Hadoop and Spark Grep benchmarks on RPi 5B cluster.

Data size	1 GB	2 GB	4 GB	8 GB	16 GB	32 GB
Hadoop	329.7	328.9	324.5	304.5	292.9	275.3
Spark	147.5	323.6	263.5	340.1	514.6	265.8

**Fig. 9:**
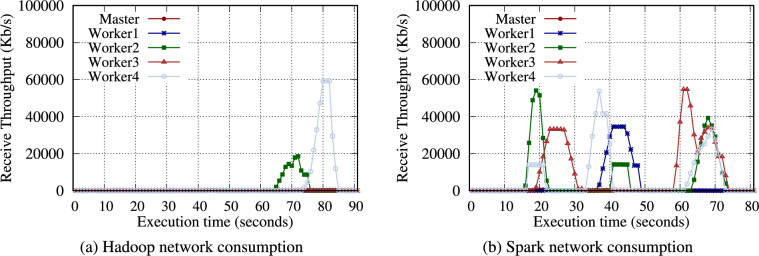
Network consumption for each node under Grep benchmark on an RPi 5B cluster with four worker nodes.

#### Grep

The Grep benchmark evaluates the performance of determining regular expressions or string patterns in extensive data. Hadoop Grep reads the input files and searches each line for the specified pattern during the map phase. The matched lines are transformed into intermediate data as key-value pairs. These intermediate data are passed to the reducer and stored in the final output file. Spark Grep reads the input file to create an RDD. Then, the filter operation checks each line for the specified pattern and filters out only matching lines. Finally, the results are saved to a file.

In Table 14, only the filtered data are written as intermediate data. Thus, the volume of intermediate data is minimal, meaning the amount of data transferred over the network is also small; hence, the duration of communication between nodes is relatively short (Fig. 9). Consequently, Grep is less affected by storage performance than WordCount or TeraSort.

**Fig. 10:**
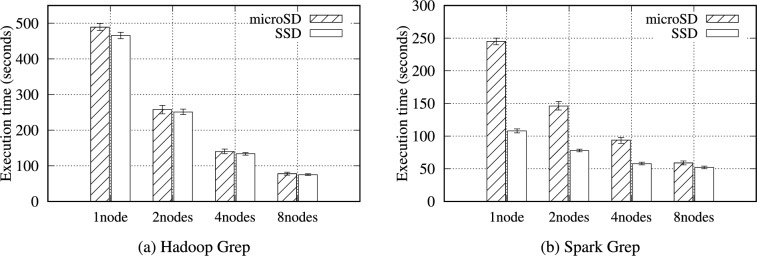
Execution time for the Hadoop and Spark Grep benchmarks with a 16GB data size on the RPi 5B cluster.

Figure 10 illustrates the total execution time of the Hadoop and Spark Grep benchmarks. Interestingly, performance gap between the SSD and microSD in Spark is greater than that in Hadoop. The final results of Hadoop Grep correspond to the number of matching lines, whereas Spark Grep stores the content of the matching lines as the final results. Thus, the performance difference is 1.16$\times$ (in Hadoop) and 2.27$\times$ on a single node with 16 GB of data.

Tables S30 and S31 present the Grep benchmark results for Hadoop and Spark. The performance gap between the SSD and microSD decreases as the node count increases because the data volume each node processes is gradually reduced. For a small data size (i.e., 1 or 2 GB), the performance does not improve as the number of nodes increases in Spark because the network data transfer time is required. Even before the SSD is not fully utilized, search processing with a small data size quickly completes.

**Table 15:** Intermediate data volume (MB) generated for the Hadoop and Spark Pi computation benchmarks on RPi 5B cluster.

Platform	Intermediate data
Hadoop (100 maps 10K samples)	115.4
Spark (10K samples)	19.7

**Fig. 11:**
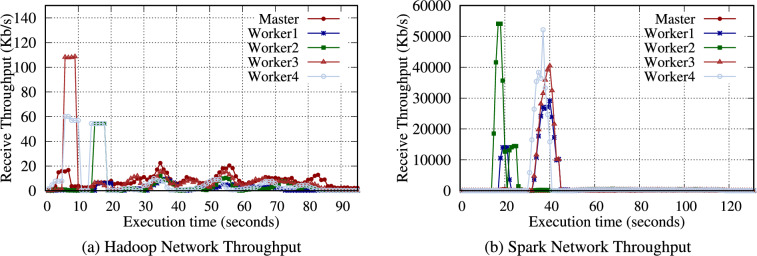
Data throughput received by each node when running the Pi computation benchmark on an RPi 5B cluster with 4 worker nodes.

#### Pi computation

The Pi computation benchmark measures the performance of calculating Pi ($\pi$) on a distributed system. This benchmark typically applies probabilistic algorithms, such as the Monte Carlo method, to estimate the value of Pi. The benchmark focuses on pure computational performance rather than data input and output, which is well-suited for testing the CPU capabilities of a system. Hadoop and Spark employ the Monte Carlo method to estimate the Pi value. The Monte Carlo method generates many random samples and applies a statistical analysis to approximate the complex mathematical problem.

The Pi computation sets up a circle inscribed within a square and generates numerous random points in the square. These points have (*x, y*) coordinates, each chosen randomly in the square bounds. The equation for a circle, $x^2 + y^2 \le r^2$, is checked to determine each point (*x, y*) exists inside the circle. Finally, the number of points inside the circle and the total number of points are counted, and these values are used to estimate Pi. Hadoop and Spark generate the assigned points in the map phase and determine whether the points are inside the circle. In the reduce phase, Hadoop and Spark aggregate the number of points calculated by each mapper or partition to estimate Pi. Table 15 and Fig. 11 reveal that the Pi computation has a high computational workload; thus, minimal intermediate data are generated, and very few data are transferred between nodes.

**Table 16:** Hadoop Pi computation benchmark results (seconds) on the RPi 5B cluster.

Configurations	Storage media	1 node	2 nodes	4 nodes	8 nodes
100 maps 10K samples	microSD	249	132	78	50
	SSD	246	131	76	49

**Table 17:** Spark Pi computation benchmark results (seconds) on the RPi 5B cluster.

Configurations	Storage media	1 node	2 nodes	4 nodes	8 nodes
10K Samples	microSD	318	169	101	74
	SSD	303	168	97	70

**Fig. 12:**
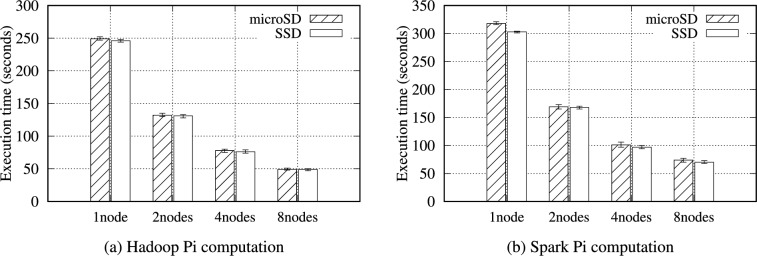
Execution time for the Hadoop and Spark Pi computation benchmarks on the RPi 5B cluster.

In Tables 16 and 17, for the Pi computation benchmark, we employed 100 maps that each use 10,000 samples per map for Hadoop and 10,000 samples for Spark. Figure 12 shows the Pi benchmark results for Hadoop and Spark on the RPi 5B cluster. This benchmark is totally CPU-intensive; hence, there is little difference in performance for each storage media, less than 1.06$\times$. As the number of nodes increases, the performance also improves accordingly due to the total CPU count increment.

**Table 18:** Hadoop TestDFSIO benchmark results on eight nodes in the RPi 5B cluster, including the throughput (MB/s), average I/O rate (MB/s), and execution time (seconds).

Operation	Data size	Throughput		Avg. I/O rate		Execution time	
		microSD	SSD	microSD	SSD	microSD	SSD
Read	1 GB	29.74	49.29	32.87	61.91	77.82	60.65
	2 GB	26.04	37.97	27.03	59.44	124.27	113.84
	4 GB	24.89	37.37	25.14	55.64	212.75	167.58
	8 GB	21.19	33.36	21.66	89.68	494.36	419.4
	16 GB	24.58	48.18	24.81	115.16	773.14	589.92
	32 GB	23.1	38.74	23.13	92.23	1,537.96	1,212.82
Write	1 GB	7.16	11.81	7.18	12.06	178.48	131.6
	2 GB	7.49	12.01	7.96	12.45	363.94	241.76
	4 GB	7.29	10.37	7.58	10.59	734.5	478.73
	8 GB	6.6	12.08	6.78	12.14	1,440.29	771.72
	16 GB	6.86	11.77	7.25	12.08	3,048.15	1,656.88
	32 GB	7.34	11.94	8.96	12.08	6,283.87	3,093.66

#### TestDFSIO

The benchmark TestDFSIO evaluates the I/O performance of the HDFS, primarily testing read and write operations. The TestDFSIO write operation creates files of a given size and writes data to the HDFS. Users can specify the file size and count. The benchmark measures the performance of writing data to the file system by creating the specified number of files. The TestDFSIO read operation reads the data from the files stored in the HDFS. The operation measures the read performance when the files are distributed across multiple nodes.

This benchmark is performed on the RPi 5B cluster with eight nodes, using 10 files. File sizes vary from 1 to 32 GB. Three primary performance metrics, throughput, average I/O rate, and execution time, are measured to evaluate the cluster I/O performance. If the throughput is lower than the average I/O rate, it indicates that the overhead, such as the network and CPU, affects the overall benchmark execution process.

**Table 19:** Spark TestDFSIO results on the 8 nodes RPi 5B cluster, consisting of throughput (MB/s), average I/O rate (MB/s), and execution time (seconds) values.

Operation	Data size	Throughput		Avg. I/O rate		Execution time	
		microSD	SSD	microSD	SSD	microSD	SSD
Read	1 GB	56.02	60.14	70.92	116.92	46.8	45.28
	2 GB	42.01	94.96	43.92	239.97	78.95	65.95
	4 GB	36.09	125.52	37.66	274.1	149.39	95.38
	8 GB	32	79.97	32.51	208.22	305.6	221.79
	16 GB	29.91	158.03	30.2	301.07	601.2	260.59
	32 GB	28.51	161.64	29.36	322.84	1,341.77	607.53
Write	1 GB	10.16	13.05	10.7	13.38	132.12	100.83
	2 GB	9.74	12.93	9.9	12.96	259.5	177.49
	4 GB	8.99	12.97	9.09	13.01	501.17	343.48
	8 GB	8.87	11.96	8.91	11.97	994.33	712.62
	16 GB	8.71	11.85	8.76	11.86	2,053.88	1,424.45
	32 GB	9.41	11.76	11.25	11.76	5,490.01	2,832.31

Tables 18 and 19 present TestDFSIO performance results of Hadoop and Spark. As the data size increases, the performance gap (i.e., the execution time difference) between the microSD and NVMe SSD also increases by an average of up to 2$\times$ at 32 GB. Regarding the relationship between the throughput and average I/O rate, Hadoop and Spark displayed similar values for writes. This means that no bottlenecks occur in the cluster when writing data. For reads, the throughput and average I/O values were similar under the microSD, whereas the performance gap noticeably increased to 2.38$\times$ for Hadoop and 2$\times$ for Spark under the NVMe SSD with a data size of 32 GB. This result indicates that other performance bottlenecks exist in the cluster. For networks, none of the benchmarks used even one-tenth of the 1 Gbps network bandwidth.

However, for all benchmarks, the microSD exhibited a very high I/O wait percentage, whereas the NVMe SSD had a very low I/O wait (e.g., see Fig. 8). Hence, the SSD was fully used by the CPU, indicating that the difference between throughput and average I/O rate is caused by the CPU. Therefore, when adopting a fast SSD as the storage media, better CPU performance accelerates big data processing further. Section Discussion discusses overclocking the CPU in more detail.

### Raspberry Pi 4B Cluster vs. 5B Cluster

The individual performance of the RPi 4B and 5B was evaluated in Section Individual Raspberry Pi performance. This section explores how individual node performance influences cluster performance. An RPi 4B cluster of five nodes (one master and four worker nodes) was built with the same settings as in the RPi 5B cluster. The microSD (Samsung PRO Plus) was adopted as the storage media because RPi 4B initially provided only a microSD slot. For a fair evaluation, we employed a newly upgraded RPi 4B with more memory (8 GB) and a more powerful CPU (1.8 GHz) than the originally released RPi 4B with up to 4 GB of RAM and a 1.5 GHz CPU.

**Fig. 13:**
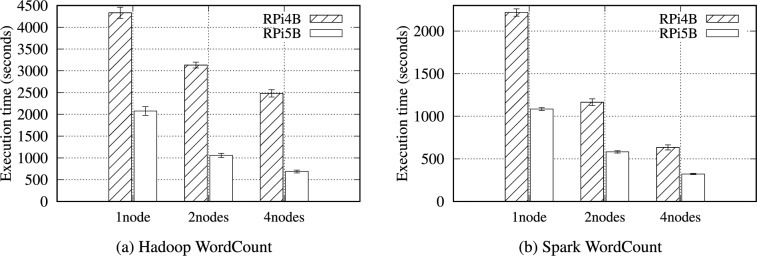
Execution time for the Hadoop and Spark WordCount benchmarks with a 16GB data size on the RPi 4B and RPi 5B Clusters.

Tables S32 and S33 list the results of the WordCount benchmark on the RPi 4B and 5B clusters. The performance gap between the RPi 4B and 5B clusters ranges from 2.2$\times$ to 3.86$\times$ under Hadoop, and from 1.85$\times$ to 2.33$\times$ under Spark. Each cluster-level performance gap noticeably increases, considering that the individual CPU performance difference corresponds to an average of 1.74$\times$ because the CPU performance significantly influences overall storage I/O performance as well as computing capability (Table 6).

Figure 13 indicates the total execution time of the RPi 4B and 5B clusters under Hadoop and Spark WordCount. As the node count increases, the performance gap between the RPi 4B and 5B clusters widens under Hadoop compared to Spark because Hadoop WordCount generates more I/O operations, implying that the storage performance is more crucial to Hadoop.

**Table 20:** Specifications of the desktop computer and RPi 5B.

	Desktop computer	RPi 5B
CPU	Intel i5-14500	ARM Cortex-A76
	@ 4.5 GHz (14 cores)	@ 2.4 GHz (4 cores)
Hyper threading	8 out of 14 cores	No support
RAM	32 GB DDR5	8 GB LPDDR4X
Ethernet	RTL8125 2.5 GbE	1 GbE RJ45 Jack
Storage media	SK Hynix GOLD P31 500 GB M.2 SSD (PCIe 3.0 $\times$ 4)	
PCIe	PCIe 4.0 (16 GT/s) $\times$ 4	PCIe 3.0 (8 GT/s) $\times$ 1

### RPi 5B cluster vs. Desktop computer

This section compares the RPi 5B cluster to a desktop computer in terms of big data processing performance and power efficiency. Table 20 lists the specifications of the desktop computer, a *modern* (not old) and *powerful* computer at a current price point of about $1,000. Unlike RPi 5B, which uses a single lane of the PCIe interface, this computer provides four PCIe lanes. Thus, this computer demonstrates unparalleled I/O performance with an average of 3.52$$\times$$ faster sequential reads and 3.35$$\times$$ faster sequential writes with a 16 MB record size (Table S34).

**Fig. 14:**
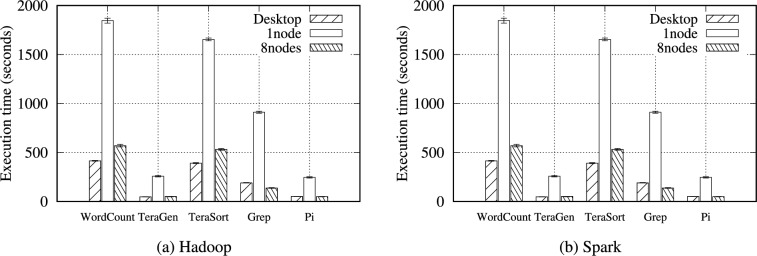
Execution time for the Hadoop and Spark benchmarks with 32 GB of data on single-node, eight-node RPi 5B clusters, and a desktop computer.

Figure 14 depicts the performance of the Hadoop and Spark benchmarks with 32 GB of data on a singe node RPi 5B, eight-node RPi 5B cluster, and a desktop computer. Under the Hadoop benchmark, the desktop computer tends to perform better under I/O-intensive workloads because it vastly benefits from the exceptional SSD I/O performance due to the four PCIe lanes. For instance, the computer performed an average of 1.37$\times$ (WordCount), 1.06$\times$ (TeraGen), and 1.36$\times$ (TeraSort) better than the RPi 5B cluster. In contrast, the RPi 5B cluster performed better under CPU-intensive workloads by taking advantage of a higher CPU core count (32 cores in the cluster): 1.37$\times$ (Grep) and 1.04$\times$ (Pi). In Spark, the RPi 5B cluster can benefit from the abundant memory space (64 GB of RAM) of the cluster in addition to the total CPU core count. Thus, Spark performed more competitively, comparable to the latest powerful computer. If the next generation RPi has a more improved I/O performance by increasing the PCIe lane count or upgrading the PCIe version, this eight-node SBC cluster is expected to defeat this single desktop computer under every workload.

**Table 21:** Total power consumption (watts) on eight-node RPi 5B cluster and a desktop PC for each benchmark.

Platform	Cluster	WordCount	TeraGen	TeraSort	Grep	Pi
Hadoop	Desktop	112.7	120	110.8	120	100
	8 $\times$ RPi 5B	64.52	62.5	80	76.33	76.2
Spark	Desktop	112.3	143.9	109.8	85.5	84.7
	8 $\times$ RPi 5B	69.93	64.2	84.75	51.81	76.75

Power consumption was also measured for the five benchmarks (Table 21). The eight-node RPi 5B cluster consumed an average of 1.52$\times$ and 1.71$\times$ less power under Hadoop and Spark, respectively, than the desktop computer. Table S35 shows the results of performance per watt for each benchmark between the desktop computer and the RPi 5B cluster. Performance was derived from the throughput per second based on size and execution time. Performance per watt was calculated as follows:

$$\begin{aligned} \text {Performance per watt} = \frac{\text {Size (GB)} / \text {Execution time (s)}}{\text {Power Consumption (W)}} \end{aligned}$$

For the performance per watt, the cluster achieved up to 2.52$\times$ better power efficiency than the desktop computer.

## Discussion

This section discusses various challenges affecting RPi 5B performance. Further, this section offers suggestions and insight into the potential performance of future RPi models.

### PCIe Version: 2.0 vs. 3.0

**Table 22:** SSD I/O performance (MB/s) comparison for PCIe 2.0 and 3.0 on RPi 5B.

Storage media	PCIe	Record size	Read (seq.)	Write (seq.)	Read (rand.)	Write (rand.)
SK Hynix GOLD P31	2.0	4 KB	154.97	112.47	64.18	141.00
		512 KB	426.60	399.35	405.31	402.73
		16 MB	446.50	417.15	447.39	413.44
	3.0	4 KB	202.23	133.73	69.45	174.00
		512 KB	797.46	742.19	717.90	751.40
		16 MB	869.47	814.13	872.12	801.67

Initially, RPi 5B supported PCIe 2.0 by default. Though PCIe 3.0 is not officially certified by the RPi Foundation, it is easily enabled with boot configuration settings by adding *dtparam=nvme* to */boot/firmware/config.txt* to enable the PCIe interface, and adding *dtparam=pciex1_gen=3* below it to switch the default from PCIe 2.0 to 3.0. To investigate the effect of PCIe versions, storage performance was measured using *iozone*. Theoretically, PCIe 2.0 and 3.0 provide a bandwidth of 5 GT/s (500 MB/s) and 8 GT/s (about 985 MB/s) per lane, respectively.

Table 22 presents the sequential read performance of the NVMe SSD at 446.5 MB/s (PCIe 2.0) and 869.47 MB/s (PCIe 3.0), respectively. Considering the internal protocol overhead of the SSD, each performance appears very reasonable. However, Hadoop TeraSort (1.01$\times$) and Spark TeraSort (1.06$\times$) benchmarks did not display a noticeable performance discrepancy between PCIe 2.0 and 3.0, considering 1.95$\times$ performance difference, primarily due to the CPU bottleneck of RPi. For verification, the CPU usage of Hadoop TeraSort was measured and reached nearly 100%. This suggests that a future RPi with a more powerful CPU would be able to utilize powerful storage media fully, such as the NVMe SSD. Section CPU Overclocking addresses this problem by overclocking the CPU of RPi 5B.

### TRIM command

For each identical experiment, a difference in performance of up to 3$\times$ was observed. This problem originates from the characteristics of the NAND flash memory-based SSD. Unlike HDDs, SSDs cannot directly overwrite data^30,31^. A garbage collection process is required to reclaim invalid (i.e., garbage) data blocks, leading to additional read and write operations and degrading performance significantly^14,32^.

Even if the data are deleted from the file system, the SSD does not recognize that the data were deleted. The OS provides a special mechanism, called a trim command, for SSDs to resolve this problem. The trim command informs the SSD that the data blocks were deleted from the system, allowing the SSD to be aware of unnecessary (i.e., deleted) blocks in advance to improve performance. In Ubuntu 23.10, the *fstrim* command is set to be called once a week by default.

Tables 11 and 13 in Section Raspberry Pi cluster performance present the characteristics of the WordCount and TeraSort workloads, generating numerous intermediate data exceeding 100 GB for the 32 GB of input data. Intermediate data are deleted after the corresponding job completes, generating a significant volume of garbage data in the SSD for each experiment. Thus, the SSD triggers an expensive garbage collection operation when the SSD reaches a predefined threshold. Therefore, the trim command, such as *fstrim* in Linux, must be executed for each benchmark with the SSD storage media to ensure that the SSD remains in an optimal state. Otherwise, the SSD may perform inconsistently in each benchmark execution.

**Table 23:** CPU performance (events per second) at 2.4 and 3.0 GHz on RPi 5B.

Threads	1	2	4	8	16
2.4 GHz	24,699.51	49,408.40	98,699.69	98,741.23	98,690.85
3.0 GHz	30,920.29	61,652.75	122,976.61	123,040.20	123,274.62

**Fig. 15:**
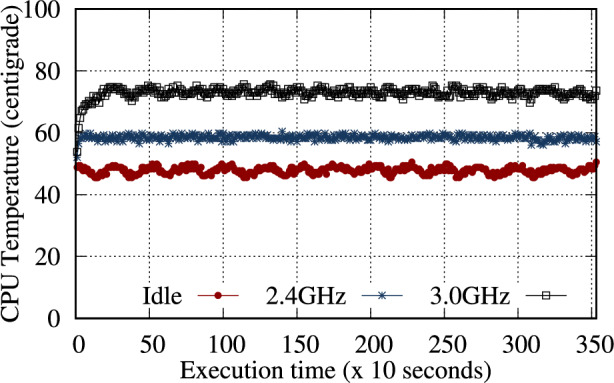
Results of CPU temperature changes in idle and stress modes at 2.4 and 3.0 GHz.

### CPU overclocking

**Fig. 16:**
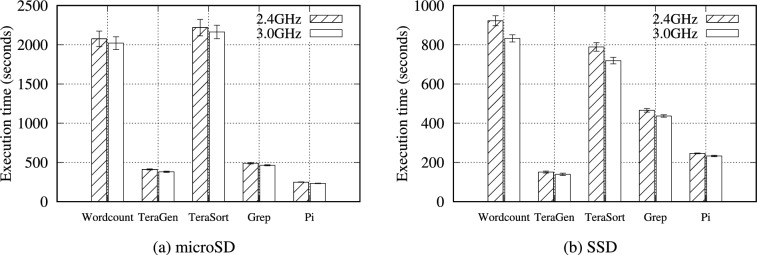
Execution time of Hadoop benchmarks with a 16GB data size on a single RPi 5B at 2.4GHz and 3.0GHz.

The CPU of RPi 5 can be overclocked from 2.4 to 3.0 GHz by adding *arm_freq=3000* and *over_voltage_delta=50000* to */boot/firmware/config.txt*. However, not all RPi 5B models can be successfully overclocked. Based on our experiments, only four out of nine overclocked RPi 5B models were operated correctly; the others failed to boot because of the silicon lottery^33^.

The overclocked CPU performance was measured using *sysbench*, and finding an average improvement of 1.25$\times$ (Table 23). The CPU temperature is also crucial for a stable CPU performance. The RPi 5B model requires an active cooling system, such as a cooling fan. A 400% CPU load was assigned for 1 hour *(stress –cpu 4 –timeout 3600)*, and the temperature change was monitored. Figure 15 presents the CPU temperature over time. At idle, the temperature remained around an average of 47.87$^{\circ }$C. At 2.4 GHz, the temperature increased to an average of 58.43$^{\circ }$C degrees, and at 3.0 GHz, it averaged 72.9$^{\circ }$C (Fig. 15).

In Table S36, all benchmarks performed better at 3.0 GHz. However, as illustrated in Fig. 16, the NVMe SSD performance improved more than that of the microSD by an average of up to 1.11$\times$. As mentioned in Section PCIe Version: 2.0 vs. 3.0, the NVMe SSD was not fully utilized due to the CPU performance bottleneck. The overclocked CPU can provide improved CPU capabilities so that it can better employ the powerful NVMe SSD. Consequently, both the Hadoop and Spark benchmarks benefit from improved CPU performance. In contrast, a slow storage media, such as microSD, benefits less from this overclocked CPU because a significantly longer CPU I/O wait time is a primary performance bottleneck, originating from the lower I/O performance of the microSD card (Fig. 8).

**Fig. 17:**
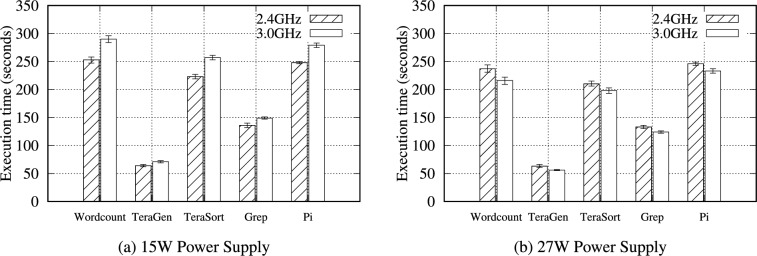
Execution time for the Hadoop benchmark with 4 GB of data on a single RPi 5B at 2.4 and 3.0 GHz, using 15 W and 27 W power supplies.

### Power Supply

We neglect the importance of the power supply; however, a sufficient power supply is essential. The RPi Foundation sells a dedicated power supply (27 W) for the RPi 5B. To investigate the influence of various power supplies, three typical power supplies (12.5, 15, and 27 W) were connected to a single RPi 5B. Using the 12.5 W power supply, the RPi 5B shut down while processing big data. The 15 W power supply successfully ran each benchmark, and each benchmark with a 3.0 GHz CPU clock performed noticeably lower than those using 2.4 GHz (Fig. 17). Moreover, all benchmarks with a 15 W power supply did not perform as well as those with a 27 W power supply, especially for all Hadoop benchmarks (Table S37).

**Fig. 18:**
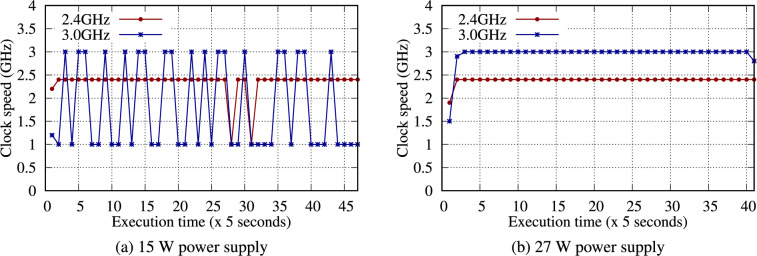
Clock speed for the Hadoop TeraSort benchmark with 4 GB of data on a single RPi 5B at 2.4 and 3.0 GHz using 15 and 27 W power supplies.

To solve this problem, we investigated each CPU clock over time for each power supply. Figure 18 illustrates a CPU clock speed under the Hadoop TeraSort benchmark. Very unstable (i.e., fluctuating) CPU clock speeds were observed under the 15 W power supply because of the power shortage for the overclocked CPU (Fig. 18-(a)), degrading performance. Conversely, the 27 W power supply provides sufficient power to the CPU so that the CPU clocks are very stable over time at full speed (Fig. 18-(b)).

### Massive dataset and parallel processing

This section explores the possibility of using RPi 5B clusters to process terabyte-scale big data. To verify this possibility, two extensive data sizes (1 and 2 TB) were employed, and three benchmarks (WordCount, TeraSort, and Grep) were evaluated on the RPi 5B cluster. For 1 TB processing, WordCount took 8,662 seconds, TeraSort took 5,311 seconds, and Grep took 773 seconds. For 2 TB processing, Grep took 1,582 seconds. However, WordCount and TeraSort failed to complete because of the insufficient total storage space (4 TB) in the cluster, not because of any computational capability problem. Both WordCount and TeraSort produce a significant volume of intermediate data, whereas Grep generates fewer intermediate data. Thus, if more storage space were provided to the RPi 5B cluster, WordCount and TeraSort benchmarks would successfully finish, with an expected 2$\times$ longer execution time similar to the Grep benchmark.

Under the real-world big data processing environment, multiple job application processing is crucial. To evaluate the current processing capabilities of the RPi 5B cluster, we ran four Spark benchmarks (i.e., WordCount, TeraSort, Grep, and Pi) simultaneously with a data size of 4 GB. The cluster successfully finished all jobs, taking 134 seconds. For a more objective comparison, the same four benchmarks were executed sequentially, taking 254 seconds. Parallel processing achieved an average of 1.9$\times$ faster execution time.

In summary, if more RPi 5B nodes were added to the cluster, it would allow a much larger dataset and more concurrent applications to be processed successfully, verifying the scalability and practicability of the RPi 5B cluster for terabyte-scale big data processing.

## Conclusion

This paper extensively examined the possibilities of SBCs for real-world big data processing by adopting the most powerful, latest generation RPi 5B. The RPi 5B model is the first generation of RPi to provide a PCIe interface that enables powerful modern storage media, such as the NVMe SSD, to directly connect to the SBC node via an external HAT board. Thus, the I/O performance of the storage media in RPi 5B has dramatically improved compared to the fastest microSD card, by an average of 9.88$\times$ for sequential reads and 15.6$\times$ for sequential writes. Further, the importance of the PCIe interface lies in the potential for expansion, including a faster network card or more powerful GPU installation. The CPU computational capability of RPi 5B has also noticeably improved by an average of up to 2.03$\times$ at the cost of 1.4$\times$ more power consumption compared to RPi 4B.

We built an RPi 5B cluster with one master node and eight worker nodes and evaluated six representative Hadoop and Spark benchmarks (WordCount, TeraGen, TeraSort, Grep, Pi computation, and TestDFSIO) to assess the cluster performance. A faster storage device, such as the NVMe SSD, was employed to evaluate the storage media performance influence on the SBC Hadoop cluster, improving the overall cluster performance by up to 3.43$\times$ compared to the current fastest microSD card. The latest RPi 5B cluster performed up to 3.86$\times$ faster than the RPi 4B cluster under the Hadoop benchmarks. The performance gap at the cluster-level is noticeably more significant than that of the CPU, considering that the individual CPU performance gap between RPi 5B and 4B (with 8 GB of RAM) is an average of 1.74$\times$ each because the CPU performance also significantly affects the overall storage I/O performance and computing capability. The RPi 5B cluster with eight worker nodes performs comparably to the desktop computer. For instance, the desktop computer tends to perform better under I/O-intensive workloads due to the 4$\times$ higher throughput of the NVMe SSD. In contrast, the RPi 5B cluster performed better under CPU-intensive workloads by utilizing more CPU cores. Regarding the performance-per-watt, the power efficiency of the cluster was up to 2.52$\times$ better than that of the desktop computer.

In addition, this paper discusses the diverse challenges and makes suggestions. The I/O bottleneck problem of the RPi was finally resolved using the PCIe interface of RPi 5B. However, based on this study, although the CPU of RPi 5B was upgraded noticeably (2.03$\times$ faster than the previous RPis), the CPU performance has become a bottleneck to exploiting the high performance storage media (i.e., NVMe SSD) fully. The CPU overclocking experiments verified this limitation. We also found that a sufficient power supply in the RPi is essential for stable, full-speed performance, preventing CPU clock fluctuations. Finally, the RPi 5B Hadoop cluster demonstrated its practicality in real-world big data fields by effectively and efficiently processing terabytes of data. The data size is no longer a limitation because RPi 5B exhibited very stable performance without system hangs, unlike the previous generations.

In summary, the PCIe interface of the RPi 5B is exceptionally beneficial. Previous generation RPis had critical limits for real-world big data processing, primarily due to the storage I/O bottleneck or insufficient memory space. Other I/O interfaces, such as microSD card slots or USB ports, cannot provide sufficient I/O throughput to accommodate massive volumes of data appropriately. The RPi 5B model also meets another critical requirement for cluster–scalability. The RPi 5B Hadoop cluster effectively expanded the processing capabilities in terms of computing and storage as the RPi 5B node count increased (i.e., scale-out). Significant advancements combined with the powerful RPi 5B and a fast PCIe-based SSD offer ’real’ possibilities in small, terabyte-scale big data processing fields.

Despite the considerable improvements, several limitations emerged under I/O-intensive workloads. To process larger volumes of data more quickly, the following aspects need to be improved. First, CPU performance and core count need to be increased. Switching the storage media from microSD to SSD resolved the previous I/O bottleneck, but under I/O-intensive workloads the CPU itself started to act as the bottleneck. Second, the SSD is not being utilized to its full potential. If a higher PCIe version were adopted or additional lanes were provided, it would be possible to extract even more performance from the storage device. Third, memory capacity is a limiting factor. In this experiment, an RPi 5B with 8 GB of memory was used. In Spark, increasing data volume can lead to a higher rate of disk swapping when memory is insufficient. A larger memory capacity would likely yield better Spark benchmark results. Through this experiment, we found that SBCs still have significant potential for further advancement in the field of big data processing.

## Supplementary Information

Supplementary Information.

## Data Availability

The datasets generated and/or analyzed during the current study are available in the Harvard Dataverse repository, 10.7910/DVN/HG7NV7.
