# Supplementary material for: Exploring the effects and potential of unlocked I/O-powered single board computer clusters
Source: Sci Rep. 2026 Jan 7;16:4486. doi: 10.1038/s41598-025-34623-x (PMC12864792; doi:10.1038/s41598-025-34623-x)
Supplement: Supplementary file 1 — Supplementary Information. [file 41598_2025_34623_MOESM1_ESM.pdf]

Table S24. Hadoop WordCount benchmark results (seconds) on the RPi 5B cluster.

Data size	Storage media	1 node	2 nodes	4 nodes	8 nodes
1 GB	microSD	106	65	58	46
	SSD	82	55	45	40
2 GB	microSD	195	124	65	62
	SSD	129	88	64	58
4 GB	microSD	429	225	189	115
	SSD	237	140	116	88
8 GB	microSD	913	460	300	214
	SSD	460	265	209	191
16 GB	microSD	2,076	1,058	688	527
	SSD	923	535	395	360
32 GB	microSD	4,444	3,069	2,398	1,243
	SSD	1,846	1,059	699	570

Table S25. Spark WordCount benchmark results (seconds) on the RPi 5B cluster.

Data size	Storage media	1 node	2 nodes	4 nodes	8 nodes
1 GB	microSD	120	86	69	67
	SSD	114	83	62	60
2 GB	microSD	171	124	77	65
	SSD	159	107	75	63
4 GB	microSD	316	178	110	78
	SSD	276	160	109	77
8 GB	microSD	579	303	183	117
	SSD	524	290	169	116
16 GB	microSD	1,085	582	321	187
	SSD	982	525	288	176
32 GB	microSD	2,113	1,099	589	327
	SSD	1,951	1,009	538	302

Table S26. Hadoop TeraGen benchmark results (seconds) on the RPi 5B cluster.

Data size	Storage media	1 node	2 nodes	4 nodes	8 nodes
1 GB	microSD	43	30	21	17
	SSD	42	26	18	15
2 GB	microSD	55	35	22	18
	SSD	45	29	20	16
4 GB	microSD	102	51	30	20
	SSD	63	36	24	19
8 GB	microSD	197	88	44	26
	SSD	91	47	29	23
16 GB	microSD	411	166	84	40
	SSD	151	74	40	30
32 GB	microSD	773	319	159	78
	SSD	257	123	64	50

Table S27. Spark TeraGen benchmark results (seconds) on the RPi 5B cluster.

Data size	Storage media	1 node	2 nodes	4 nodes	8 nodes
1 GB	microSD	33	32	31	31
	SSD	29	29	29	29
2 GB	microSD	50	37	37	37
	SSD	34	33	32	32
4 GB	microSD	91	57	44	44
	SSD	47	41	39	39
8 GB	microSD	175	98	67	55
	SSD	75	57	46	44
16 GB	microSD	338	184	126	84
	SSD	127	85	68	59
32 GB	microSD	666	349	235	138
	SSD	236	134	103	73

Table S28. Hadoop TeraSort benchmark results (seconds) on the RPi 5B cluster.

Data size	Storage media	1 node	2 nodes	4 nodes	8 nodes
1 GB	microSD	98	77	58	51
	SSD	76	56	52	46
2 GB	microSD	196	133	94	81
	SSD	131	80	73	70
4 GB	microSD	423	194	135	125
	SSD	210	136	121	79
8 GB	microSD	891	460	274	245
	SSD	387	228	169	155
16 GB	microSD	2,219	1,558	779	612
	SSD	789	463	427	311
32 GB	microSD	5,357	2,636	1,740	1,449
	SSD	1,654	919	688	532

Table S29. Spark TeraSort benchmark results (seconds) on the RPi 5B cluster.

Data size	Storage media	1 node	2 nodes	4 nodes	8 nodes
1 GB	microSD	69	62	54	49
	SSD	52	49	47	46
2 GB	microSD	125	81	66	60
	SSD	85	69	57	52
4 GB	microSD	287	147	94	81
	SSD	171	115	79	70
8 GB	microSD	561	275	147	104
	SSD	312	191	117	93
16 GB	microSD	1,153	532	283	159
	SSD	619	340	205	134
32 GB	microSD	3,001	1,112	588	287
	SSD	1,160	614	346	211

Table S30. Hadoop Grep benchmark results (seconds) on the RPi 5B cluster.

Data size	Storage media	1 node	2 nodes	4 nodes	8 nodes
1 GB	microSD	45	30	25	21
	SSD	43	29	24	21
2 GB	microSD	79	45	37	26
	SSD	73	44	32	24
4 GB	microSD	139	78	52	38
	SSD	133	77	46	33
8 GB	microSD	258	141	80	47
	SSD	249	133	79	44
16 GB	microSD	489	258	140	78
	SSD	466	251	134	75
32 GB	microSD	922	500	262	139
	SSD	911	477	256	137

Table S31. Spark Grep benchmark results (seconds) on the RPi 5B cluster.

Data size	Storage media	1 node	2 nodes	4 nodes	8 nodes
1 GB	microSD	39	34	33	33
	SSD	27	27	28	29
2 GB	microSD	51	41	39	38
	SSD	31	30	31	33
4 GB	microSD	76	68	54	46
	SSD	43	40	35	37
8 GB	microSD	129	92	64	48
	SSD	65	53	42	41
16 GB	microSD	245	146	94	59
	SSD	108	78	58	52
32 GB	microSD	441	258	144	96
	SSD	196	123	80	67

Table S32. Hadoop WordCount benchmark results (seconds) on the RPi 4B and 5B clusters with microSD.

Data size	RPi model	1 node	2 nodes	4 nodes
1 GB	RPi 4B	247	149	121
	RPi 5B	106	65	58
2 GB	RPi 4B	445	291	208
	RPi 5B	195	124	65
4 GB	RPi 4B	978	648	540
	RPi 5B	429	225	189
8 GB	RPi 4B	2,012	1,276	1,145
	RPi 5B	913	460	300
16 GB	RPi 4B	4,333	3,133	2,482
	RPi 5B	2,076	1,058	688
32 GB	RPi 4B	9,792	6,982	6,005
	RPi 5B	4,444	3,069	2,398

Table S33. Spark WordCount benchmark results (seconds) on the RPi 4B and 5B clusters with microSD.

Data size	RPi model	1 node	2 nodes	4 nodes
1 GB	RPi 4B	267	200	149
	RPi 5B	120	86	69
2 GB	RPi 4B	359	230	165
	RPi 5B	171	124	77
4 GB	RPi 4B	607	364	234
	RPi 5B	316	178	110
8 GB	RPi 4B	1,163	649	376
	RPi 5B	579	303	183
16 GB	RPi 4B	2,218	1,165	634
	RPi 5B	1,085	582	321
32 GB	RPi 4B	4,341	2,232	1,185
	RPi 5B	2,113	1,099	589

Table S34. SSD I/O performance (MB/s) on a desktop computer and RPi 5B.

Storage media	Node	Record size	Read (seq.)	Write (seq.)	Read (rand.)	Write (rand.)
SK Hynix GOLD P31	Desktop	4 KB	396.36	110.97	53.69	108.15
		512 KB	2,187.22	2,009.34	1,615.47	2,360.82
		16 MB	3,059.28	2,728.43	3,167.61	3,227.62
	RPi 5B	4 KB	201.95	134.01	69.48	174.42
		512 KB	798.30	742.66	717.13	751.87
		16 MB	869.80	814.47	873.48	800.23

Table S35. Performance per watt (MB/W) on eight-node RPi 5B cluster and a desktop PC for each benchmark.

Platform	Cluster	WordCount	TeraGen	TeraSort	Grep	Pi
Hadoop	Desktop	0.68	5.68	0.74	1.42	6.27
	8 × RPi 5B	0.87	10.24	0.75	3.06	8.60
Spark	Desktop	0.60	4.48	0.90	8.32	4.11
	8 × RPi 5B	1.51	6.83	1.79	9.21	5.95

Table S36. Total execution time of various Hadoop and Spark benchmarks (seconds) with a 16GB of data on a single RPi 5B at 2.4GHz and 3.0GHz.

Storage	Platform	GHz	WordCount	TeraGen	TeraSort	Grep	Pi
microSD	Hadoop	2.4	2,076	411	2,219	489	249
		3.0	2,022	381	2,163	464	233
	Spark	2.4	1,085	338	1,153	245	318
		3.0	995	335	1,125	235	253
SSD	Hadoop	2.4	923	151	789	466	246
		3.0	833	139	719	437	233
	Spark	2.4	982	127	619	108	303
		3.0	891	125	561	103	246

Table S37. Total execution time for Hadoop and Spark benchmarks (seconds) with 4 GB of data on the SSD. We ran these benchmarks on a single RPi 5B using 15 and 27 W power supplies.

Watts	Platform	GHz	WordCount	TeraGen	TeraSort	Grep	Pi
15W	Hadoop	2.4	253	64	223	136	248
		3.0	290	71	257	149	279
	Spark	2.4	277	51	172	43	304
		3.0	264	51	187	46	251
27W	Hadoop	2.4	237	63	210	133	246
		3.0	216	56	198	124	233
	Spark	2.4	276	47	171	43	303
		3.0	216	45	161	40	246
